# The effect of medical innovation on the cost-effectiveness of Covid 19-related policies in the United States using a SIR model

**DOI:** 10.1186/s12913-023-09282-1

**Published:** 2023-04-18

**Authors:** Adam J. Atherly, Eline M. van den Broek-Altenburg

**Affiliations:** 1 Virginia Commonwealth University, Richmond, VA 23284 USA; 2 University of Vermont Larner College of Medicine, Burlington, VT 05405 USA

**Keywords:** Covid-19, Cost-effectiveness, Non-medical interventions, Vaccines, Susceptible-Infected-Recovered model, Incremental cost effectiveness

## Abstract

**Background:**

During 2020–21, the United States used a multifaceted approach to control SARS-CoV-2 (Covid-19) and reduce mortality and morbidity. This included non-medical interventions (NMIs), aggressive vaccine development and deployment, and research into more effective approaches to medically treat Covid-19. Each approach had both costs and benefits. The objective of this study was to calculate the Incremental Cost Effectiveness Ratio (ICER) for three major Covid-19 policies: NMIs, vaccine development and deployment (Vaccines), and therapeutics and care improvements within the hospital setting (HTCI).

**Methods:**

To simulate the number of QALYs lost per scenario, we developed a multi-risk Susceptible-Infected-Recovered (SIR) model where infection and fatality rates vary between regions. We use a two equation SIR model. The first equation represents changes in the number of infections and is a function of the susceptible population, the infection rate and the recovery rate. The second equation shows the changes in the susceptible population as people recover. Key costs included loss of economic productivity, reduced future earnings due to educational closures, inpatient spending and the cost of vaccine development. Benefits included reductions in Covid-19 related deaths, which were offset in some models by additional cancer deaths due to care delays.

**Results:**

The largest cost is the reduction in economic output associated with NMI ($1.7 trillion); the second most significant cost is the educational shutdowns, with estimated reduced lifetime earnings of $523B. The total estimated cost of vaccine development is $55B. HTCI had the lowest cost per QALY gained vs “do nothing” with a cost of $2,089 per QALY gained. Vaccines cost $34,777 per QALY gained in isolation, while NMIs alone were dominated by other options. HTCI alone dominated most alternatives, except the combination of HTCI and Vaccines ($58,528 per QALY gained) and HTCI, Vaccines and NMIs ($3.4 m per QALY gained).

**Conclusions:**

HTCI was the most cost effective and was well justified under any standard cost effectiveness threshold. The cost per QALY gained for vaccine development, either alone or in concert with other approaches, is well within the standard for cost effectiveness. NMIs reduced deaths and saved QALYs, but the cost per QALY gained is well outside the usual accepted limits.

**Supplementary Information:**

The online version contains supplementary material available at 10.1186/s12913-023-09282-1.

## Background

During the majority of 2020 and 2021, the United States used a multifaceted approach to control SARS-CoV-2 (Covid-19) and reduce mortality and morbidity from the disease. This included a series of non-medical interventions (NMIs), aggressive vaccine development and deployment, and research into more effective approaches to medically treat Covid-19. Each of the different approaches had both costs and benefits.

NMIs were the most visible and controversial approach and generally fell into one of two categories: 1. measures to limit the physical contact of individuals and 2. measures to reduce the probability of transmission per individual. NMIs in the first category included measures such as stay-at-home orders, the closing of schools, cancellation of public events, restrictions on social gatherings, closing public transportation, travel restrictions, and closing of nonessential businesses. NMIs in the second category included measures such as the requirement to wear masks in public, tracking and tracing, and increased testing [1]. During April of 2020, state and local policies required almost 90% of the total population to stay at home unless engaged in “essential” activities; after that time, state and local restrictions have waxed and waned in response to fluctuations in local Covid-19 rates. These measures to limit physical contact led to a sharp economic recession [2–6]. The unemployment rate went from one of the lowest levels since records have been kept to the highest rate since the Great Depression – with the change occurring essentially in a single month [7].

At the same time, the Federal government initiated “Operation Warp Speed” to rapidly develop and deploy a vaccine for Covid-19 [8]. Although there was skepticism about the ability to develop, test, and deploy a vaccine in under a year [9], biopharmaceutical companies developed three vaccines which were approved for emergency use in under 12 months. The effort and resources devoted to the development and deployment of the vaccine were unprecedented [10].

Lastly, the healthcare system rapidly repurposed existing treatments and biopharmaceutical companies developed new Covid-19 therapeutics to better treat patients in the hospital, although this was done with much less fanfare than NMIs or Vaccines. These included antivirals like Remdesivir, steroids like dexamethasone, and monoclonal antibodies [11, 12]. There were also numerous changes in clinical care, including using nasal oxygen and putting patients in the prone position. Overall, the in-hospital mortality rate decreased by 47% over the first six months of the pandemic [13].

There have been several attempts to put these policy measures into a health economic context in the United States. Early economic analyses of policy responses estimated benefits and costs but did not explicitly consider non-health outcomes [14–17]. As the pandemic progressed, studies started measuring the impact of specific NMIs on the effective reproduction number R_0_ alone or gains in terms of Quality-Adjusted Life Years (QALYs) [18–21]. Other cost–benefit analyses focused on specific health impacts such as mental health [22, 23], increases in domestic violence [24, 25], child health such as declines in routine pediatric vaccine ordering [26], deferred care in general [27], or for specific diseases such as cancer [28]. Evaluation of costs have focused on economic costs of the measures either calculated by small business closings [15], the economic stimulus package [16], Gross Domestic Product (GDP) loss [4, 14] or loss of productivity [17]. Studies documented the cost-effectiveness of individual measures such as hand washing and mask wearing, the social consequences of lockdowns, and the effect of the Covid-19 pandemic on Americans in debt [6]. Early analyses were plagued by uncertainty around the data inputs [29, 30] and did not consider the role of therapeutics and care improvements within the hospital setting or vaccinations.

This study analyzes the cost-effectiveness of three policy responses to Covid-19: NMIs focused on lockdowns, therapeutics and care improvements within the hospital setting (HTCI), and vaccine development and deployment over a 24-month period. Our analysis uses a multi-risk Susceptible-Infected-Recovered (SIR) model to predict the total number of Covid-19 related QALY losses under each policy response.

## Methods

We performed a cost-effectiveness analysis, comparing the gains in QALYs to the costs to calculate the cost per QALY gained for different policy scenarios. We then calculated an Incremental Cost-Effectiveness Ratio (ICER) showing the cost per QALYs gained between each policy and a counterfactual “do nothing” scenario. For costs and benefits, we have adopted the societal perspective.

### Cost of Covid-19 policy response

NMI costs are focused on those measures to limit the physical contact of individuals. Costs from other types of NMIs (use of masks, social distancing, testing, and tracing) are assumed to be minimal in comparison and not specifically included in the analysis. The key cost of NMIs comes from the reduction in economic output due to the closure of many businesses during the lockdowns.

The Congressional Budget Office (CBO) provides economic forecasts and data on economic performance; the July 2021 CBO projection is our baseline – this is what is expected would have happened to the economy without Covid-19 [31, 32]. This can be compared to actual economic performance – and projected future economic performance – using post Covid-19 CBO data. Presumably, some reduction in economic performance would have occurred regardless of NMIs due to consumer behavior (e.g., consumers reducing their visits to restaurants even in the absence of restrictions). We assume a baseline consumer reaction of 30% with a sensitivity analysis.

A second area of cost impact will be changes in healthcare spending related to Covid-19. Avoided cases of Covid-19 will lead to reductions in healthcare spending for the infections that did not occur. This cost of the avoided hospitalizations is included using the average cost per Covid-19 hospitalization [33] multiplied by the number of hospitalizations. There were also immediate cost savings associated with healthcare services that normally would have been delivered but were not due to Covid-19. For this analysis, we draw a distinction between deferred care – care that was delayed but ultimately delivered – and eliminated care from discretionary medical visits – care that would have been delivered but was sufficiently time sensitive that the delay led to elimination. To calculate this, we draw on estimates of deferred care in the literature [34–36]. We also include cost estimates for increased substance abuse and domestic violence [37].

We include two categories of costs associated with school closures: for the parents, who must provide childcare that would normally be provided by schools, which is based on industry specific estimates of time lost from work [38], and for children, who will have reduced labor market performance due to reduced learning. The 24.2 million children aged 5 to 11 years who attended public schools that were closed or transitioned to online learning due to the pandemic will be disadvantaged relative to previous and subsequent generations who did not have reductions in learning. Across all U.S. states, public schools were closed for a median 54.0 days as a result of Covid-19 during the spring of 2020 [39]. For the 2020–21 school year, a Centers for Disease Control and Prevention (CDC) analysis showed that only 45% of the 22.4 m children in public schools had in-person education (using a weighted average of the entire academic year) while the rest were online. The number of hours of education per week is estimated to have decreased by between 10 and 45%, with a mid-range estimate of 22% [40], suggesting a long-term labor earning decline of 1.27% [41].

### Inpatient Spending

Avoided cases of Covid-19 will lead to reductions in healthcare spending for the infections that did not occur. To calculate the hospital specific costs avoided, we first calculated Covid-19 related costs using data on the average cost per Covid-19 hospitalization by age group ($20,360), multiplied by the number of cases by age group from the CDC [33]. The number of cases that would have occurred in the different scenarios is based on the number of total Covid-19 cases multiplied by the hospitalization rate (1.5%).

### Education

The key economic impact on children will be reduced long-term labor market performance due to reduced learning. Christakis et al. found 24.2 million children aged 5 to 11 years attended public schools that were closed during the 2020 due to the pandemic and that, across all US states, public schools were closed for a median 54.0 days as a result of Covid-19 during the spring [39]. This suggests that on average children lost approximately 0.15 final years of education as a result of school closures in Spring 2020 [39]. For 2020–21, a CDC analysis showed found that only 45% of 22.4 m children in public schools had in-person education during the 2020–21 school year (using a weighted average of the entire academic year) while the rest were online. Studies from France, Italy and Germany suggest that the number of hours of education per week decreased by between 10 and 45%, with the mid-range estimate of 22% [40], which computes to an average reduction of education of approximately 0.099 academic years of education for 2020–21. The average US K-12 school has 180 days, so the combined effect of the closures (0.15) and transition to online learning (0.099) is equivalent to missing 44.8 days of school. Jaume and Willen (2019) find that an 88-day teacher strike in Argentina reduced labor earnings an average of 2.6%. Therefore, a reduction of 44.8 days should result in a long-term labor earning decline of 1.27%. Average lifetime earnings is $1.7 m [41], for a reduction in long-term earnings of $21,590 for each of 24.2 m students for a total cost of $523B.

An alternative formulation be to use the results from Psacharapoulos et al. (2018), which suggest that one additional year of schooling increased lifetime earning between 5 and 10% [42]. Using the midpoint (7.5%) and a loss of 0.249 years of education and the same lifetime wages suggests a lifetime wage decrease of $768B. For our analysis, we use the more conservative estimate of $523B.

### Other Costs

For substance abuse, published literature suggests a roughly 12% increase in substance abuse during pandemic and total substance abuse costs are estimated to be approximately 7% of GDP [37]. We use a similar technique for domestic violence. Estimates of the total cost of domestic abuse suggest that the total cost of domestic violence is equal to 3.3% of total GDP, with estimates that domestic violence has increased by 20% due to NMIs [43].

### Vaccine Development Costs

The total estimated cost of vaccine development is $55B. This includes $11.5B in vaccine development and manufacturing [44–54] plus $11.8B in vaccine purchases ($6.97B to Pfizer, $4.85B to Modern and $1B to Johnson and Johnson) [55–62]. The largest cost associated with vaccines is distribution and administration, with an estimated cost of $32B. This includes $8.8B in the December, 2020 Covid Relief Act to the CDC to support federal, state, local, territorial and tribal public health agencies distribute, administer, monitor and track coronavirus vaccination to ensure broad-based distribution, access and vaccine coverage [63] and $23.3B in the American Rescue Plan Act of 2021, which included a further $7.5B for Covid–19 vaccine distribution and administration, including support for State, local, Tribal, and territorial public health departments, $1B for vaccine confidence, information, and education activities, $6.1B to support the supply chain for Covid-19 vaccines, therapeutics, and ancillary medical products through research, development, manufacturing, production, and purchasing, $500 m to the FDA for activities related to Covid-19 vaccines, therapeutics, and diagnostics, including for evaluation of their continued performance, safety, and effectiveness and facilitation of advanced continuous manufacturing, $7.6 to HHS for community health centers for activities including COVID-19 vaccine distribution and administration, testing, contact tracing, mitigation, workforce enhancement, and community outreach and education and $600 m to the Indian Health Service for Covid-19 vaccine distribution and administration.

### Hospital therapeutics and Care Improvements

For therapeutic development, the total cost of studying ($85 M), manufacturing ($450 m) and contracting ($98 m) Regeneron was $633 m [46, 64–66]. The cost for AstraZeneca monoclonal antibody development and manufacturing was $458 m [67] and the cost for Merck MK-7110 development and manufacturing was $356 million [68]. There were also a number of less expensive treatments, suggesting a total cost for therapeutic development of $1.8B [69–73]. The cost of therapeutic purchase is estimated at $6.2B, including $2.6B for Regeneron and $1.6B for Remdesivir [74–80].

### QALYs gained

To simulate the number of QALYs lost per scenario, we developed a multi-risk Susceptible-Infected-Recovered (SIR) model where infection and fatality rates vary between regions. The model was proposed by Kermack et al. [81]; the version we use is similar to Teulings (2021), which mimics Acemoglu et al. [82, 83].

We use a two equation SIR model. The first equation represents changes in the number of infections I for period i and is a function of the susceptible population (S), the infection rate (β) and the recovery rate (γ). The second equation shows the changes in the susceptible population as people recover. The recovery rate is the inverse of the infection fatality rate. Our model enables a tractable quantitative analysis of optimal policy similar to those already developed in the context of the homogeneous-agent SIR models.

1 $$\Delta I_i=\beta S_i-\gamma I_i$$

2 $$\Delta S_i=-I_i$$

Vaccines have the effect of reducing the susceptible population, modifying Eq. (2) to:

3 $$\Delta S_i=-I_i-V_i$$

**Fig. 1:**
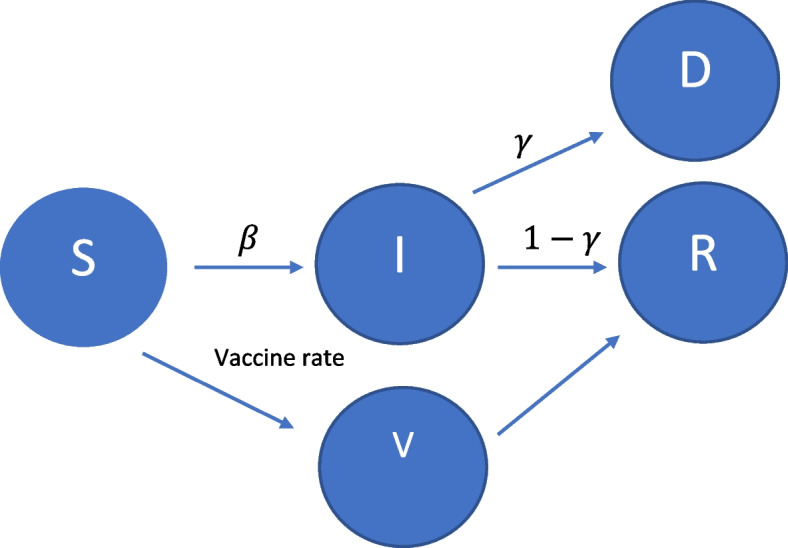
Graphical Depiction of SIR Model

With V showing the number of individuals vaccinated in time period i. The model is graphically depicted in Fig. 1.

The model was calibrated to actual death rates. For the initial values, the calibrations parameter β was set to 0.05, with adjustments for warmer weather which then transitions regionally to the colder weather β in the fall/ winter. The initial infected fatality rate was set at 0.5%, then reduced to 0.4% in the summer and 0.2%in the winter of 2020/21 [12]. The susceptible population was set to 330 million and divided into six different “regions”.

NMIs, HTCIs and vaccinations are introduced into the SIR model during the same period they were implemented. The simulations are based on the effectiveness of different interventions in modifying the different parameters. On average, a Covid-19 death leads to an average QALY loss of 7.65 at the median age of death [84].

Each of the different interventions had a different intended purpose. The purpose of NMIs is to reduce the number of infected individuals. Although the precise impact of NMIs on the rate of infection is a source of considerable controversy, evidence from the peer-review literature suggests that NMIs collectively reduce the rate of infection by 17% [85].

The purpose of HTCIs is to reduce the mortality rate for infected individuals but not the infection rate. In the first three months of the pandemic (March–May 2020), 2.95% of hospitalized cases reported to the CDC died [33]. By the next three-month period (June–August 2020), the fatality rate had dropped to 2.07%, a 43% drop. This drop is a function of new therapeutics, including the use of Remdesivir, which was found to reduce 29-day mortality rates by approximately 28% [86] and, more recently, Remdesivir plus Baricitinib, which reduces in-hospital mortality by 53% [87]. Our overall Infection Fatality Rate (IFR) is 0.5%, which we reduce to 0.4% after June 2020 to reflect the initial hospital therapeutics and care improvements and then to 0.2% in October 2020 [88].

Finally, vaccines prevent individuals from becoming infected. For the vaccines, we use data on the proportion of the population that has been vaccinated by date and then extrapolate to herd immunity when 75% of the population is either vaccinated or has been infected [89]. When a person is vaccinated, they are removed from the susceptible population for the SIR model.

For the analysis we included a number of different scenarios. These included the “do nothing” alternative, which represents the number of QALY losses that would have occurred if no action had been taken in response to Covid-19. This is not intended to necessarily represent a realistic alternative, but rather a consistent benchmark to compare with each of the scenarios. We then vary the infection rate to model NMIs, the fatality rate to model HTCIs, and the susceptible population to model vaccines.

### QALY losses due to indirect health effects of policy measures

Other than direct QALY gains from the policy measures, there are also unintended consequences of the policies in terms of QALY losses. These include deaths due to care deferred due to health system shutdowns, increases in suicides and other mental health problems, increases in substance abuse, domestic violence, child abuse and neglect, and increases in preventable childhood illnesses.

To date, there has been no complete assessment of all avoided care, deferred care, delayed care and care not delivered in the United States. There are two U.S.-based studies that show a reduction in service delivery, but neither include nationally representative data [27, 90], and a study from the United Kingdom that estimates the effect of the delays on increased mortality in the U.K. [91].

For this analysis, we focused on excess cancer deaths due to Covid-19. Lai et al. (2020) report that in the United States there was a 45–66% decrease in chemotherapy treatment due to Covid-19 related measures and a 70–89% decrease in referrals for screening [28]. Maringe et al. (2020) found that delayed diagnosis due to the Covid-19 measures over a 12-month duration led to an increase of 7.9–9.6% mortality among breast cancer patients within 5 years, a 15.3–16.6% increase in mortality among colon cancer patients, a 4.8–5.3% increase among lung cancer patients and a 5.8–6.0% increase among esophageal cancer [92].

## Results

### Costs

Table 1 shows the costs associated with the different policy options. The largest cost is the reduction in economic output associated with NMI lockdowns. The difference in CBO projections covering fiscal 2020 and 2021 is $1.7 trillion [93–95]. The second most significant cost is the educational shutdowns associated with NMIs, with estimated reduced lifetime earnings of $523B. Other NMI-related costs, which includes domestic violence and child abuse, substance abuse treatment and additional childcare for parents, with savings from deferred care, was calculated at $110B (see details in the Appendix). Hospitalization costs for Covid-19 were significant but varied little across scenarios ($59B-$48B). The total estimated cost of vaccine development is $55B [44–63]. The estimated total cost of HTCIs was $8.0B [46, 64–80].

**Table 1:** Costs by policy option

	Do Nothing	NMIs Only	Hospital Improvements Only	Vaccines Only	NMIs + Hospital Improvements	NMIs + Vaccines	Hospital Improvements + Vaccines	NMIs + Hospital Improvements + Vaccines
2-year GDP Loss	$500B	$1.7 T	$500B	$500B	$1.7 T	$1.7 T	$500B	$1.7 T
Hospitalization Costs	$59B	$59B	$59B	$52B	$52B	$48B	$52B	$48B
Other NMI Related Economic Costs	$0	$110B	$0	$0	$110B	$110B	$0	$110B
Educational Shutdowns	$0	$523B	$0	$0	$523B	$523B	$0B	$523B
Therapeutic Development	$0	$0	$8.0B	$0	$8.0B	$0	$8.0B	$8.0B
Vaccine Development	$0	$0	$0	$55B	$0	$55B	$55B	$55B
Total Cost	**$559B**	**$2.39 T**	**$567B**	**$607B**	**$2.39 T**	**$2.44 T**	**$615B**	**$2.45 T**

*NMI* Non-Medical Interventions; table reflects the economic costs of different policy options and combinations of policy options by major cost category

### QALY gains and losses

**Fig. 2:**
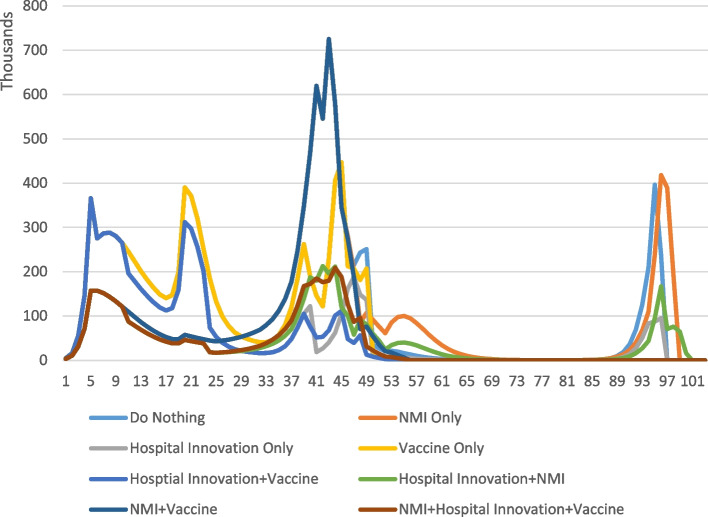
Number of predicted QALY losses by week by policy option. Results based on the SIR model with the number of weekly QALY losses under different policy scenarios for weeks 1–101 of the pandemic

Total QALYs lost under the five modeled scenarios are presented in Table 2 and Fig. 2. The “do nothing” approach is similar in total QALYs lost *due to Covid-19* (10.27 m) to the NMIs-only approach (9.96 m); this is because without any HTCIs or vaccines, the only effect of NMIs is to delay deaths. Although discounting means that a later death is more valuable, the QALY value of brief delays is negligible. However, scenarios with NMIs also result in additional QALY losses due to deferred care. An average excess mortality of 10% of the 1.8 million new cancer patients would lead to 180,000 excess deaths in the next 5 years, with average QALY loss of 10.64, or 1,915,200 QALYs lost due to increased cancer mortality. QALY losses are also relatively high under the vaccine-only policy – 8.89 m QALYs lost. Models with HTCIs show much lower QALY losses – 6.44 m without NMIs and 6.72 m with NMIs (but no vaccine) and 5.62 m with vaccines (but no NMIs).

**Table 2:** Net Quality-Adjusted Life Years (QALY) Lost Under Different Policies

	**Do Nothing**	**NMI Only**	**Hospital Improvements Only**	**Vaccine Only**	**Hospital Improvements + Vaccine**	**Vaccine + NMI**	**Hospital Improvements + NMI**	**Hospital Improvements + NMI + Vaccine**
**QALYs lost due to Covid-19**	10.27 m	9.96 m	6.44 m	8.89 m	5.62 m	7.54 m	4.80 m	3.97 m
**Deferred Care (cancers)**	–	1.92 m	–	–	–	1.92 m	1.92 m	1.92 m
**Net QALYs Lost**	10.27 m	11.88 m	6.44 m	8.89 m	5.62 m	9.46 m	6.72 m	5.89 m

Table reflects the number of QALY losses from different policy options and combinations of policy options by major category

Table 3 takes the costs (from Table 1) and benefits (from Table 2) and calculates the cost per QALY gained for each of the scenarios compared to the base cases of “do nothing” and hospital therapeutics and care improvements alone. The NMI-only approach delayed deaths due to Covid-19 but created additional deaths through deferred care, while incurring costs of shutting down the economy. The most cost-effective approach was HTCIs – $2,089 per QALY gained. This was followed by HTCIs plus vaccines ($15,773) and vaccines only ($34,777), which were also cost effective.

**Table 3:** 24-Month Cost-Effectiveness Ratios

		*Do Nothing*	*Hospital Improvements Only*	*NMIs Only*	*Vaccines Only*	*NMIs* + *Hospital Improvements*	*NMIs* + *Vaccines*	*Hospital Improvements* + *Vaccines*	*NMIs* + *Hospital Improvements* + *Vaccines*
*Total Cost*		$559B	$567B	$2.39 T	$607B	$2.39 T	$2.44 T	$615B	$2.45 T
*QALYs Lost*		10.27 m	6.44 m	11.88 m	8.89 m	5.62 m	9.46 m	6.72 m	5.89 m
Compared to “Do Nothing”	Incremental Cost	Reference	$8B	$1,831B	$48B	$1,839B	$1,881B	$56B	$1,882B
	Incremental QALYs gained	Reference	3.8 m	-1.61 m	1.4 m	4.7 m	0.8 m	3.6 m	4.4 m
	Cost per QALY Gained	–	$2,089	Dom	$34,777	$391,277	$2,351,250	$15,773	$427,727
Compared to “Hospital therapeutics and Care Improvements Only”	Incremental Cost	–	Reference	$1,823 T	$40.0B	$1,831B	$1,873B	$56B	$1,874B
	Incremental QALYs Gained	–	Reference	-5.4 m	-2.5 m	-280 k	-3.0 m	820 k	550 k
	Cost per QALY Gained	–	–	Dom	Dom	Dom	Dom	$58,528	$3.4 m

*Dom* Dominated. Table shows the Incremental Cost Effectiveness Ratio (ICER) based on a “Do Nothing” reference category and Hospital Therapeutics and Care Improvements alone. Dominated categories have both higher QALY losses and higher costs

HTCIs as a base case dominates most of the alternatives, including NMIs only, vaccines only, NMIs and HTCIs, and NMIs with vaccines. Adding vaccines to HTCIs reduced QALYs and increased costs, with a net cost of $58,528 per QALY gained. Adding both vaccines and NMIs also reduced mortality and increased costs, with a net cost of $3.4 m per QALY gained.

**Fig. 3:**
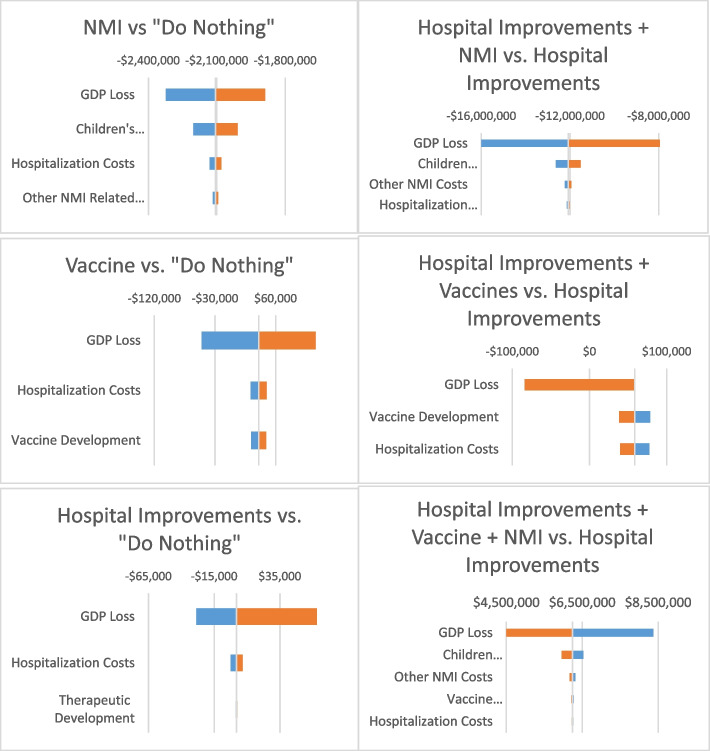
Tornado diagrams showing sensitivity to key cost inputs

Figure 3 shows the sensitivity of the results to the different cost inputs. All alternatives were most sensitive to the estimated GDP losses, which were generally given a range of ± 30%, which is equivalent to assuming either no consumer reaction to Covid-19 to 60% of the decrease would have happened regardless of policy. NMIs were dominated throughout (i.e., both higher costs and lower QALYs gained). Vaccines ranged from $119,650 per QALY saved to being cost saving (if consumer reaction was reduced due to the presence and promise of a vaccine). Similarly, using the base of HTCIs, NMIs are dominated, vaccines range from cost effective ($77,529 per QALY gained) to cost saving and the combination of vaccines and NMIs saves QALYs, but at a very high cost per QALY gained (ranging from over $8 m per QALY gained to $4 m per QALY gained).

## Discussion

This paper examined the costs and benefits of different policies enacted to combat Covid-19. After more than two years of the pandemic, we can now look backwards at the cost effectiveness of different policies to help understand which policies should be prioritized and avoided in the future. We focus on three different broad groups of interventions: NMIs, HTCIs, and vaccines. In general, all options that incorporate NMIs have a high cost per QALY saved, HTCIs have a very small cost per QALY gained, and vaccines are well within the standard benchmarks for cost effectiveness. This strength of this paper is its combination of a SIR model with a cost-effectiveness analysis. This allows a consideration of the costs and benefits of different policies during the duration of the pandemic, rather than using an arbitrary time cut-off. To our knowledge, no previous study of Covid-19 has used this approach.

There is a clear logic to the results. NMIs alone do not prevent any deaths – they simply delay infections and thus delay deaths. Indeed, lockdowns used in isolation (without HTCIs or vaccines) are worse than the “do nothing” option because they do not prevent Covid-19 deaths but do lead to unintended deaths. NMI lockdowns are also the most expensive option, with costs running into the trillions of dollars. The combination of high costs, unintended deaths, and inability to independently prevent Covid-19 deaths leads to not only a high cost-effectiveness ratio for NMIs, but also for any interventions that are combined with NMIs.

Vaccines are the most effective alternative in preventing Covid-19, but even with the unprecedented speed of vaccine development, the vaccines still arrived after more than 400,000 deaths had occurred, limiting the number of QALYs gained. There was a surge of deaths both in the spring of 2020 and then a second surge in the winter of 2020–2021, which impacted much of the population prior to the development of vaccines. However, even with the extraordinary cost associated with the “warp speed” development and the year long delay in development and deployment, vaccines remain very cost effective at about $35 k per QALY gained as a stand-alone intervention.

The most cost-effective intervention was HTCIs. This was due in large part to the ability to repurpose therapeutics developed against other diseases, such as antivirals developed against MERS and West Nile virus, monoclonal antibodies used in immune disorders, and steroids used for other respiratory conditions. It was also due to the ability to rapidly develop new antibody therapeutics and to adapt clinical practice, such as changing patients’ position, which did not lead to direct cost increases but reduced mortality meaningfully.

There are several limitations to this analysis. Due to lack of data elements, we had to exclude many variables from the cost-effectiveness analysis. These include mental, physical and nutritional effects of school closures, the effect on adults extended time at home, such as weight gain, suicides and more. We also focused our NMI analysis on lockdowns and did not fully consider the costs or benefits of PPE usage, testing, social distancing, or other NMI efforts. While other studies have shown these interventions to be cost-effective [41], our rationale from a societal perspective was that the costs of these interventions were minimal compared to GDP losses and school closings.

The counterfactual of consumer reaction may be a limitation. We assumed that without state and local lockdowns, consumer activity still would have declined by 30% in our baseline model. A smaller consumer reaction would mean NMIs would be even less cost effective, but a larger consumer reaction would have reduced the cost effectiveness ratios of NMIs. However, as shown in Exhibit 4, even if the consumer reaction in the absence of NMI lockdowns was 60% of the actual reaction – suggesting extremely risk-averse consumers – NMIs would still be dominated.

We also use population averages, rather than age adjusted rates. This shouldn’t create bias in the results because Covid-19 policies were not age-specific. The exception to this was the age specific eligibility for vaccines. Removing higher risk persons from the population first via vaccination could lead to a reduction in the mortality rate within the remaining population, which would mean that vaccines are somewhat more cost-effective than our model suggests. The age limitations were short-lived, however, so the effect will be negligible.

This study also lends support to current CDC guidelines, which recommend against lockdowns or school closures except in unusual situations, such as hospital capacity limitations. These guidelines reduce two of the key unintended consequences of NMIs – increased deaths from cancer and productivity losses due to reduced educational attainment, by keeping schools and medical facilities open, while also avoiding the significant GDP losses associated with business closures.

The cost effectiveness of the different policies also depends on the timing of the other policies. NMIs are more cost effective when coupled with HTCIs and vaccines. Using NMIs to delay infections early in the pandemic long enough for first HTCIs and then vaccines to be developed and deployed can potentially be justified under a cost effectiveness framework. However, absent future clinical developments or hospital capacity issues, current or future efforts to delay infections will have an extremely high cost per QALY gained. These may be helpful policy messages towards future pandemic preparedness.

## Supplementary Information

**Additional file 1.**

## Data Availability

The datasets used and/or analyzed during the current study are available from the corresponding author on reasonable request.
