# Supplementary material for: The effect of medical innovation on the cost-effectiveness of Covid 19-related policies in the United States using a SIR model
Source: BMC Health Serv Res. 2023 Apr 18;23:372. doi: 10.1186/s12913-023-09282-1 (PMC10111306; doi:10.1186/s12913-023-09282-1)
Supplement: Supplementary file 1 — Additional file 1. [file 12913_2023_9282_MOESM1_ESM.docx]

**Appendix: Cost Calculations**

*Economic Closures:* The Congressional Budget Office (CBO) provides economic forecasts and data on economic performance; the July 2021 CBO projection is our baseline – this is what is expected would have happened to the economy without Covid-19[1, 2]. This can be compared to actual economic performance – and projected future economic performance – using post Covid-19 CBO data. Presumably, some reduction in economic performance would have occurred regardless of NMIs due to consumer behavior (e.g., consumers reducing their visits to restaurants even in the absence of restrictions). We assume a baseline consumer reaction of 30% with a sensitivity analysis.

*Healthcare Spending:* Avoided cases of Covid-19 will lead to reductions in healthcare spending for the infections that did not occur. This cost of the avoided hospitalizations is included using the average cost per Covid-19 hospitalization[3] multiplied by the number of hospitalizations. There were also immediate cost savings associated with healthcare services that normally would have been delivered but were not due to Covid-19. For this analysis, we draw a distinction between deferred care – care that was delayed but ultimately delivered – and eliminated care from discretionary medical visits – care that would have been delivered but was sufficiently time sensitive that the delay led to elimination. To calculate this, we draw on estimates of deferred care in the literature[4-6]. We also include cost estimates for increased substance abuse and domestic violence[7].

*Inpatient Spending:* Avoided cases of Covid-19 will lead to reductions in healthcare spending for the infections that did not occur. To calculate the hospital specific costs avoided, we first calculated Covid-19 related costs using data on the average cost per Covid-19 hospitalization by age group ($20,360), multiplied by the number of cases by age group from the CDC [3]. The number of cases that would have occurred in the different scenarios is based on the number of total Covid-19 cases multiplied by the hospitalization rate (1.5%).

*Education:* Christakis et al found 24.2 million children aged 5 to 11 years attended public schools that were closed during the 2020 due to the pandemic and that, across all US states, public schools were closed for a median 54.0 days as a result of Covid-19 during the spring[8]. This suggests that on average children lost approximately 0.15 final years of education as a result of school closures in Spring 2020 [8]. For 2020-21, a CDC analysis showed found that only 45% of 22.4m children in public schools had in-person education during the 2020-21 school year (using a weighted average of the entire academic year) while the rest were online. Studies from France, Italy and Germany suggest that the number of hours of education per week decreased by between 10% and 45%, with the mid-range estimate of 22%[9], which computes to an average reduction of education of approximately 0.099 academic years of education for 2020-21. The average US K-12 school has 180 days, so the combined effect of the closures (0.15) and transition to online learning (0.099) is equivalent to missing 44.8 days of school. Jaume and Willen (2019) find that an 88-day teacher strike in Argentina reduced labor earnings an average of 2.6%. Therefore, a reduction of 44.8 days should result in a long-term labor earning decline of 1.27%.

An alternative formulation be to use the results from Psacharapoulos et al (2018), which suggest that one additional year of schooling increased lifetime earning between 5 and 10%[10]. Using the midpoint (7.5%) and a loss of 0.249 years of education and the same lifetime wages suggests a lifetime wage decrease of $768B. For our analysis, we use the more conservative estimate of $523B.

*Vaccine Development and Deployment Costs*: The total estimated cost of vaccine development is $55B. This includes $11.5B in vaccine development and manufacturing [11-21] plus $11.8B in vaccine purchases ($6.97B to Pfizer, $4.85B to Modern and $1B to Johnson and Johnson). [22-29] The largest cost associated with vaccines is distribution and administration, with an estimated cost of $32B. This includes $8.8B in the December, 2020 Covid Relief Act to the CDC to support federal, state, local, territorial and tribal public health agencies distribute, administer, monitor and track coronavirus vaccination to ensure broad-based distribution, access and vaccine coverage[30] and $23.3B in the American Rescue Plan Act of 2021, which included a further $7.5B for Covid–19 vaccine distribution and administration, including support for State, local, Tribal, and territorial public health departments, $1B for vaccine confidence, information, and education activities, $6.1B to support the supply chain for Covid-19 vaccines, therapeutics, and ancillary medical products through research, development, manufacturing, production, and purchasing, $500m to the FDA for activities related to Covid-19 vaccines, therapeutics, and diagnostics, including for evaluation of their continued performance, safety, and effectiveness and facilitation of advanced continuous manufacturing, $7.6 to HHS for community health centers for activities including COVID-19 vaccine distribution and administration, testing, contact tracing, mitigation, workforce enhancement, and community outreach and education and $600m to the Indian Health Service for Covid-19 vaccine distribution and administration.

*Hospital therapeutics and Care Improvements*: For therapeutic development, the total cost of studying ($85M), manufacturing ($450m) and contracting ($98m) Regeneron was $633m[13, 31-33]. The cost for AstraZeneca monoclonal antibody development and manufacturing was $458m[34] and the cost for Merck MK-7110 development and manufacturing was $356 million[35]. There were also a number of less expensive treatments, suggesting a total cost for therapeutic development of $1.8B[36-40]. The cost of therapeutic purchase is estimated at $6.2B, including $2.6B for Regeneron and $1.6B for Remdesivir[41-47].

*Other Costs:* We also included the costs associated with school closures for the parents, who must provide childcare that would normally be provided by schools, which is based on industry specific estimates of time lost from work and wage rate[48]. For substance abuse, published literature suggests a roughly 12% increase in substance abuse during pandemic and total substance abuse costs are estimated to be approximately 7% of GDP[20]. We use a similar technique for domestic violence. Estimates of the total cost of domestic abuse suggest that the total cost of domestic violence is equal to 3.3% of total GDP, with estimates that domestic violence has increased by 20% due to NMIs [49].

References

1. CBO, *The decline in CBO's Projections of GDP for 2020 and 2021*.

2. CBO, *Additional Information About the Economic Outlook: 2021 to 2031*. 2021.

3. CDC. *Covid Tracker*. 2020; Available from: <https://covid.cdc.gov/covid-data-tracker/#datatracker-home>.

4. Fund, C., *The Impact of COVID-19 on Outpatient Visits in 2020: Visits Remained Stable, Despite a Late Surge in Cases.* February 22, 2021.

5. Rogers, H.M., Mills, C. and Kramer, M.J., *Estimating the impact of COVID-19 on healthcare costs in 2020: Key factors of the cost trajectory*, in *Milliman*. 23 April 2020.

6. Daniel McDermott , N.K., Giorlando Ramirez , Nicolas Shanosky , and Cynthia Cox Follow @cynthiaccox on Twitter, *2021 Premium Changes on ACA Exchanges and the Impact of COVID-19 on Rates*, KFF, Editor. 2021.

7. Mark, T.L., et al., *Changes In US spending on Mental Health And Substance Abuse Treatment, 1986-2005, and implications for policy.* Health Aff (Millwood), 2011. **30**(2): p. 284-92.

8. Christakis, D.A., W. Van Cleve, and F.J. Zimmerman, *Estimation of US Children’s Educational Attainment and Years of Life Lost Associated With Primary School Closures During the Coronavirus Disease 2019 Pandemic.* JAMA Network Open, 2020. **3**(11): p. e2028786-e2028786.

9. G, D.P., et al., *The likely impact of COVID-19 on education: Reflections based on the existing literature and recent international datasets*, in *JRC Technical Report*. 2020, European Commission.

10. Psacharopoulos, G. and H.A. Patrinos, *Returns to investment in education: a decennial review of the global literature.* Education Economics, 2018.**26**(5): p. 445-458.

11. CongressionalResearchOffice. *Operation Warp Speed Contracts for COVID-19 Vaccines and Ancillary Vaccination Materials*. 2020; Available from: <https://crsreports.congress.gov/product/pdf/IN/IN11560>.

12. Bloomberg. *Inside Operation Warp Speed’s $18 Billion Sprint for a Vaccine*. 2020; Available from: <https://www.bloomberg.com/news/features/2020-10-29/inside-operation-warp-speed-s-18-billion-sprint-for-a-vaccine>.

13. CommitteeResponsibleFederalBudget, *COVID Money Tracker.* 2020.

14. BusinessWire. *Moderna Announces Expansion of BARDA Agreement to Support Larger Phase 3 Program for Vaccine (mRNA-1273) Against COVID-19*. 2020; Available from: <https://www.businesswire.com/news/home/20200726005025/en/Moderna-Announces-Expansion-BARDA-Agreement-Support-Larger>.

15. Gavi. *Gavi welcomes final approval of U.S. support for global immunisation in Year-End Omnibus and COVID Supplemental Package*. 2020; Available from: <https://www.gavi.org/news/media-room/gavi-welcomes-final-approval-us-support-global-immunisation-covid-supplemental-package>.

16. HHS. *Biden Administration purchases additional doses of COVID-19 vaccines from Pfizer and Moderna*. 2020; Available from: <https://www.hhs.gov/about/news/2021/02/11/biden-administration-purchases-additional-doses-covid-19-vaccines-from-pfizer-and-moderna.html>.

17. HHS. *HHS DOD collaborate plasn purchase Lilly*. 2020; Available from: <https://www.hhs.gov/about/news/2020/10/28/hhs-dod-collaborate-plans-purchase-lilly-investigational-therapeutic-treat-covid-19.html>

18. HHS. *HHS DOD Collaborate with Jonhson and Johnson*. 2020; Available from: <https://www.hhs.gov/about/news/2020/08/05/hhs-dod-collaborate-with-johnson-and-johnson-to-produce-millions-of-covid-19-investigational-vaccine-doses.html>

19. HHS. *HHS DOD collaborate with Novamax*. 2020; Available from: <https://www.hhs.gov/about/news/2020/07/07/hhs-dod-collaborate-novavax-produce-millions-covid-19-investigational-vaccine-doses-commercial-scale-manufacturing-demonstration-projects.html>

20. Merck. *Merck to Help Produce Johnson & Johnson’s COVID-19 Vaccine; BARDA to Provide Merck With Funding to Expand Merck’s Manufacturing Capacity for COVID-19 Vaccines and Medicines*. 2020; Available from: <https://www.merck.com/news/merck-to-help-produce-johnson-barda-to-provide-merck-with-funding-to-expand-mercks-manufacturing-capacity-for-covid-19-vaccines-and-medicines/>.

21. MedicalCounterMeasures. *BARDA partners with Merck and IAVI to accelerate development of a vaccine to prevent COVID-19*. 2020; Available from: <https://www.medicalcountermeasures.gov/newsroom/2020/merck-iavi/>.

22. HHS. *HHS DOD Partner Sanofi GSK Commercial Scale Manufacturing*. 2020; Available from: <https://www.hhs.gov/about/news/2020/07/31/hhs-dod-partner-sanofi-gsk-commercial-scale-manufacturing-demonstration-project-produce-millions-covid-19-investigational-vaccine-doses.html>

23. HHS. *Trump Administration Accelerates AstraZeneca*. 2020; Available from: 11. <https://www.hhs.gov/about/news/2020/05/21/trump-administration-accelerates-astrazeneca-covid-19-vaccine-to-be-available-beginning-in-october.html>

24. *Rare look at Operation Warp Speed's unprecedented effort to produce, distribute COVID-19 vaccine*, C. News, Editor. 2020.

25. NCSL, *COVID-19 Economic Relief Bill*. 2021.

26. Congress. *H.R. 1319*. 2021; Available from: <https://www.congress.gov/117/bills/hr1319/BILLS-117hr1319enr.pdf>.

27. KFF. *What’s in the American Rescue Plan for COVID-19 Vaccine and Other Public Health Efforts?* 2021; Available from: <https://www.kff.org/policy-watch/whats-in-the-american-rescue-plan-for-covid-19-vaccine-and-other-public-health-efforts/>.

28. WhiteHouse. *President Biden Announces American Rescue Plan*. 2021; Available from: <https://www.whitehouse.gov/briefing-room/legislation/2021/01/20/president-biden-announces-american-rescue-plan/>.

29. Facher, L., Cohrs, R. *Biden requests $415 billion from Congress to ramp up the country’s Covid-19 response*. 2021; Available from: <https://www.statnews.com/2021/01/14/biden-request-covid-19-response/>.

30. NCSL. *COVID-19 Economic Relief Bill*. 2021; Available from: <https://www.ncsl.org/ncsl-in-dc/publications-and-resources/covid-19-economic-relief-bill-stimulus.aspx>.

31. MedicalCountermeasures. *News release from HHS.Gov: HHS, DOD collaborate with Merck to continue the development and large-scale manufacturing of investigational COVID-19 treatment*. 2020; Available from: <https://www.medicalcountermeasures.gov/newsroom/2020/merck-1/>.

32. MedicalCountermeasures. *Regeneration Study*. 2020; Available from: <https://www.medicalcountermeasures.gov/newsroom/2020/regeneron-study/>

33. Regeneron. *Regeneron Announces Manufacturing and Supply Agreement for BARDA and U.S. Department of Defense for REGN-COV2 Anti-Viral Antibody Cocktail*. 2020; Available from: <https://investor.regeneron.com/news-releases/news-release-details/regeneron-announces-manufacturing-and-supply-agreement-barda-and>.

34. HHS. *Trump Administration Expands Collaboration with AstraZeneca to Develop and Manufacture an Investigational Monoclonal Antibody to Prevent COVID-19*. 2020; Available from: <https://www.hhs.gov/about/news/2020/10/09/trump-administration-expands-collaboration-with-astrazeneca-to-develop-and-manufacture-an-investigational-monoclonal-antibody-to-prevent-covid-19.html>.

35. MedicalCounterMeasures. *Regeneration Therapeutic*. 2020; Available from: <https://www.medicalcountermeasures.gov/newsroom/2020/regeneron-therapeutic/>.

36. Johnson&Johnson. *Johnson & Johnson to Expand Partnership with U.S. Department of Health & Human Services to Accelerate the Discovery of Potential COVID-19 Treatments*. 2021; Available from: <https://www.jnj.com/johnson-johnson-to-expand-partnership-with-u-s-department-of-health-human-services-to-accelerate-the-discovery-of-potential-covid-19-treatments>.

37. MedicalCountermeasures. *BARDA and Genentech expand partnership to begin Phase 2 clinical trial to evaluate investigational treatments for COVID-19 patients*. 2021; Available from: <https://www.medicalcountermeasures.gov/newsroom/2020/genentech-therapeutic/>.

38. MedicalCountermeasures. *BARDA leverages CIADM Partner for Development of Plasma-based Therapeutic Treatment for COVID-19*. 2021; Available from: <https://www.medicalcountermeasures.gov/newsroom/2020/emergent-plasma/>.

39. SAbBiotherapeutics. *SAB-185: Advancing Novel Polyclonal Therapeutic for COVID-19*. 2020; Available from: <https://www.sabbiotherapeutics.com/covid-19/>.

40. MedicalCountermeasures. *HHS, Department of Defense, and Grifols Collaborate to Develop Plasma-based Treatment for COVID-19*. 2021; Available from: <https://www.medicalcountermeasures.gov/newsroom/2020/grilfols-plasma/>.

41. HHS. *Trump AdministrationPurchases Additonal 100 million Doses Covid-19 Investigationel Vaccine Moderna*. 2020; Available from: <https://www.hhs.gov/about/news/2020/12/11/trump-administration-purchases-additional-100-million-doses-covid-19-investigational-vaccine-moderna.html>

42. HHS. *Trump administration secures new supplies remdesivir United States*. 2020; Available from: <https://www.hhs.gov/about/news/2020/06/29/trump-administration-secures-new-supplies-remdesivir-united-states.html>

43. Regeneron. *Regeneron Announces U.S. Government Agreement to Purchase Additional COVID-19 Antibody Cocktail Doses*. 2021; Available from: <https://newsroom.regeneron.com/news-releases/news-release-details/regeneron-announces-us-government-agreement-purchase-additional>.

44. StatNews. *Gilead announces long-awaited price for Covid-19 drug remdesivir*. 2020; Available from: <https://www.statnews.com/2020/06/29/gilead-announces-remdesivir-price-covid-19/>.

45. Lilly. *Lilly announces 650,000 additional doses of neutralizing antibody bamlanivimab (LY-CoV555) purchased by U.S. government to treat COVID-19*. 2020; Available from: <https://investor.lilly.com/news-releases/news-release-details/lilly-announces-650000-additional-doses-neutralizing-antibody>.

46. Bloomberg. *Lilly Gains After Antibody Sales Boost Quarterly Earnings*. 2021; Available from: <https://www.bloomberg.com/news/articles/2021-01-29/lilly-earnings-beat-estimates-as-covid-antibody-sales-take-off>.

47. Lilly. *Lilly announces additional doses of neutralizing antibody therapy purchased by U.S. government to treat COVID-19*. 2021; Available from: <https://investor.lilly.com/news-releases/news-release-details/lilly-announces-additional-doses-neutralizing-antibody-therapy>.

48. Strong, A. and J.W. Welburn, *An Estimation of the Economic Costs of Social-Distancing Policies*. 2020: RAND.

49. Waters, H.H., et al., *The economic dimensions of interpersonal violence.* 2004.
